# Back to the cell cycle with SAMHD1 and its viral antagonist, Vpx

**DOI:** 10.1186/1742-4690-10-S1-O26

**Published:** 2013-09-19

**Authors:** Florence Margottin-Goguet

**Affiliations:** 1 INSERM, U1016, Institut Cochin, 22 rue Méchain, Paris, 75014, France

## 

Vpx from HIV-2/SIVsm and Vpr from HIV-1 work in a similar way. Their respective functions indeed require the recruitment of the same cellular ubiquitin ligase, assembled by cullin 4A. However both viral proteins are not redundant from the functional point of view: Vpr induces cell cycle arrest at the G2 phase of the cell cycle, Vpx has no apparent effect on cell cycle progression. The specificity of their action results from their capacity to recruit different cellular targets, which are subsequently ubiquitinated and degraded. As such, SAMHD1 was identified as the cellular restriction factor specifically inactivated by Vpx, but not by HIV-1 Vpr. The Vpr target whose degradation may lead to G2 arrest is still unknown today, despite the discovery of several cellular proteins degraded in the presence of the viral protein. Among them, we identified two transcription factors, ZIP and sZIP.

Ironically, it is the study of Vpx but not that of Vpr that drives us back into the cell cycle field. Indeed, SAMHD1 is a dNTP hydrolase, which restricts HIV-1 in quiescent cells by depleting the intracellular pool of dNTP. In contrast, SAMHD1 does not restrict the virus in cycling cells where dNTP levels are abundant, reaching maximal concentrations in S-phase when cells do replicate their DNA. This led us to address the question of whether SAMHD1 controls the proliferation status of the cell.

